# The Modification of Regulatory Circuits Involved in the Control of Polyhydroxyalkanoates Metabolism to Improve Their Production

**DOI:** 10.3389/fbioe.2020.00386

**Published:** 2020-04-30

**Authors:** Claudia Velázquez-Sánchez, Guadalupe Espín, Carlos Peña, Daniel Segura

**Affiliations:** ^1^Departamento de Microbiología Molecular, Instituto de Biotecnología, Universidad Nacional Autónoma de México, Cuernavaca, Mexico; ^2^Departamento Ingeniería Celular y Biocatálisis, Instituto de Biotecnología, Universidad Nacional Autónoma de México, Cuernavaca, Mexico

**Keywords:** polyhydroxyalkanoates, global regulation, *Azotobacter vinelandii*, gene regulation, biopolymers

## Abstract

Poly-(3-hydroxyalkanoates) (PHAs) are bacterial carbon and energy storage compounds. These polymers are synthesized under conditions of nutritional imbalance, where a nutrient is growth-limiting while there is still enough carbon source in the medium. On the other side, the accumulated polymer is mobilized under conditions of nutrient accessibility or by limitation of the carbon source. Thus, it is well known that the accumulation of PHAs is affected by the availability of nutritional resources and this knowledge has been used to establish culture conditions favoring high productivities. In addition to this effect of the metabolic status on PHAs accumulation, several genetic regulatory networks have been shown to drive PHAs metabolism, so the expression of the PHAs genes is under the influence of global or specific regulators. These regulators are thought to coordinate PHAs synthesis and mobilization with the rest of bacterial physiology. While the metabolic and biochemical knowledge related to the biosynthesis of these polymers has led to the development of processes in bioreactors for high-level production and also to the establishment of strategies for metabolic engineering for the synthesis of modified biopolymers, the use of knowledge related to the regulatory circuits controlling PHAs metabolism for strain improvement is scarce. A better understanding of the genetic control systems involved could serve as the foundation for new strategies for strain modification in order to increase PHAs production or to adjust the chemical structure of these biopolymers. In this review, the regulatory systems involved in the control of PHAs metabolism are examined, with emphasis on those acting at the level of expression of the enzymes involved and their potential modification for strain improvement, both for higher titers, or manipulation of polymer properties. The case of the PHAs producer *Azotobacter vinelandii* is taken as an example of the complexity and variety of systems controlling the accumulation of these interesting polymers in response to diverse situations, many of which could be engineered to improve PHAs production.

## Introductory Remarks

Petrochemical plastics are materials widely used in industry and daily life. The excessive use of such synthetic materials poses detrimental effects on the environment because they are non-biodegradable and accumulate in the ecosystems. To deal with this problem, biodegradable plastics have emerged as an alternative to replace petrochemical plastics.

Polyhydroxyalkanoates (PHAs) are among the natural biodegradable plastics under research. These are polyesters composed by (R)-hydroxyalkanoate monomers which are synthesized by a wide number of bacterial species as a carbon and energy reserve material. Besides biodegradable, PHAs are biocompatible and structurally diverse, with more than 150 different kinds of (R)-hydroxyalkanoate monomers composing them (Steinbüchel and Lütke-Eversloh, 2003). According to the number of carbon atoms in their monomeric units, PHAs have been classified as short chain length (C4 and C5; scl-PHAs) and medium chain length (≥C6; mcl-PHAs) (Aldor and Keasling, 2003).

This variety of monomers represents an advantage over other biopolymers because it allows having diverse material properties. PHAs have the most variable melting temperatures (Tm), glass-transition temperatures (Tg), and thermodegradation temperatures (Td), with mechanical properties including a very flexible Young’s modulus, elongation at break and tensile strength (Chen, 2010; Volova et al., 2013).

The diversity of PHAs is possible because the polymerizing enzymes producing them, PHA synthases, have a broad substrate range (Stubbe et al., 2005). In addition, the substrate monomers can be synthesized from different metabolic routes, depending on the microbial species used and the carbon source provided (reviewed in Lu et al., 2009; Chen, 2010; Panchal et al., 2013).

In the case of PHB, the homopolymer composed of the C4 3-hydroxyalkanoic acid 3-hydroxybutyrate (an scl-PHA), the biosynthesis starts with two molecules of acetyl-CoA that condense, forming acetoacetyl-CoA due to the activity of 3-ketothiolase (*phbA* gene). Acetoacetyl-CoA is then reduced by an acetoacetyl-CoA reductase (encoded by *phbB*) using NADPH and producing 3-hydroxybutyryl-CoA. Finally, the 3-hydroxybutyryl-CoA monomers are polymerized by a PHA synthase (*phbC* gene) (Lenz and Marchessault, 2005).

Poly-(3-hydroxyalkanoates) composed of C6–C16 3-hydroxyalkanoic acids or mcl-PHAs, are generally produced from carbon sources structurally related to these monomers. In this case, the (R)-3-hydroxyacyl-CoAs precursor monomers come from β-oxidation of alkanes, alkanols or alkanoic acids (Kniewel et al., 2019). This requires producing the (R)-hydroxyacyl-CoA isomer (substrate for polymerization by the PHA synthase), instead of the (S)-hydroxyacyl-CoA normally produced during β–oxidation, by the activity of enzymes like the alternative enoyl-CoA hydratase encoded by *phaJ* (Tsuge et al., 2003). mcl-PHAs can also be produced from acetyl-CoA in several *Pseudomonas* species. In this case, the (R)-3-hydroxyacyl-CoAs are generated from (R)-3-hydroxyacyl-ACP intermediates of the *de novo* fatty acid biosynthesis, by the activities of an (R)-3-hydroxyacyl-ACP thioesterase (encoded by *phaG*) and an (R)-3-hydroxyacyl-CoA ligase, allowing the synthesis of mcl-PHAs from non-related carbon sources, like carbohydrates (Wang et al., 2012; Hokamura et al., 2015).

Under certain conditions, the PHAs accumulated are degraded by poly(3-hydroxyalkanoate) depolymerase enzymes (gene *phaZ*) producing biosynthetic precursors and reducing equivalents (Sznajder and Jendrossek, 2011).

Because of their biodegradability and their manufacture from renewable resources, commercial production of PHAs started in the 1980s by Imperial Chemical Industries, and later by several companies (Chen, 2009; Dietrich et al., 2017). These are used for the production of everyday items like containers, packaging films, coatings, bottles and other disposable items like razors, diapers, cups, bags, lids, etc. (Chen, 2009). Due to their biocompatibility and ability to maintain human cell growth, PHAs are also under research for many biomedical applications such as medical devices (stents, sutures, cardiovascular devices, nerve repair devices and wound dressing), tissue engineering scaffolds, drug-delivery systems, dental materials, etc. (Panchal et al., 2013).

Although the use of PHAs as plastic materials for bulk applications represents a benefit for the environment, wide utilization of these polymers at the industrial level is still limited, mainly due to their high production costs that lead to a high price in comparison to conventional plastics. PHAs can cost between United States $2.25–2.75/lb, which is 3 to 4 times higher than polymers like PE and PP (Kourmentza et al., 2017). These costs are due in part to the high price of the substrates used, complex downstream processing, and the cultivation strategies using discontinuous batch or two-step cultivation and fed-batch cultivation modes, that impact productivity (Choi and Lee, 1997; Kourmentza et al., 2017). These cultivation strategies are needed because PHAs accumulation is usually promoted when there is excess carbon in the medium, but under limitation of a nutrient essential for growth (Koller et al., 2010); therefore, a cultivation phase favoring growth must be implemented first to reach high cell density (needed for an intracellular product), followed by a growth-limiting PHAs production phase. This requirement of nutrient limitation is, in many cases, controlled by genetic regulatory mechanisms that coordinate PHAs production with the rest of the metabolism; therefore, modification of these regulatory circuits could help modify this necessity, improving productivity and as a consequence, production costs.

The existence of some bacteria able to produce PHAs during growth, with little or no requirement of growth-limiting conditions (reported for *Azotobacter vinelandii, Alcaligenes latus, Pseudomonas putida* LS46, *Methylobacterium* sp. ZP24, *Bacillus mycoides* RLJ B-017 and recombinant *Escherichia coli*) (Choi and Lee, 1997; Koller et al., 2010; Blunt et al., 2019), shows that is possible to have growth-associated production, thus enabling the implementation of more efficient cultivation strategies. Therefore, a better understanding of physiological conditions and the genetic regulatory mechanisms restricting PHAs synthesis during optimal growth could be exploited to improve productivity. Also, the advances in the knowledge of PHAs biosynthesis have led to the development of improved strains for the more efficient production of PHAs and the production of novel polymers, and also will allow the engineering of PHAs producers through the use of synthetic biology methods (Jung et al., 2010; Chen et al., 2016; Chen and Jiang, 2017, 2018). However, in these strategies, the modification of the regulatory circuits controlling PHAs synthesis is generally not considered. In the future of strain modification not only the metabolic networks but also the regulatory circuits controlling them can be optimized to achieve improved performance in PHAs production (Jung et al., 2010). For this, a better understanding of the regulatory mechanisms involved is needed.

## Regulatory Mechanisms Controlling Phas Metabolism

### Control of Enzymatic Activity

Bacteria control PHAs metabolism in response to environmental or nutritional conditions. This is done through diverse regulatory mechanisms. In *Ralstonia eutropha*, the model organism for scl-PHAs metabolism (Reinecke and Steinbüchel, 2009), the main genes involved in PHB metabolism (*phaA, phaB, phaC, phaR*, and *phaZa1*) seem to be constitutive and do not show significant changes in their transcripts levels throughout the cell cycle (Lawrence et al., 2005; Brigham et al., 2012; Sharma et al., 2016). However, one possibility of regulation is the control of enzyme activity. In fact, the first mechanisms described for the control of PHB synthesis was the allosteric regulation of the β-ketothiolase biosynthetic enzyme and of enzymes of the tricarboxylic acid cycle. Both *R*. *eutropha* (Haywood et al., 1988; Oeding and Schlegel, 1973) and *Azotobacter beijerinckii* (Senior and Dawes, 1973), showed inhibitory action by free CoA (most of it produced in the TCA cycle) on the β-ketothiolase condensation reaction, and of NAD(P)H on enzymes of the TCA cycle, proving that PHB metabolism is in part controlled by the redox state of the cell and the availability of carbon (intracellular levels of acetyl-CoA and free CoA), showing a central role for oxygen limitation in the control of PHB metabolism.

More recently, allosteric regulation was also found to participate in the control of PHAs metabolism in the mcl-PHAs producer *P*. *putida* GPo1. In this bacterium, Ren et al. (2009) analyzed the granule-bound proteins and showed that PHA polymerase (PhaC), PHA depolymerase (PhaZ) and an acyl-CoA synthetase (ACS1) are present simultaneously, and this could constitute a futile cycle. By studying enzymatic activities of some of these proteins, they found that the PhaC synthase is sensitive to the ratio of [R-3-hydroxyacyl-CoA]/[CoA], and free CoA is a moderate competitive inhibitor. On the other hand, the fatty acid oxidation complex (which is a provider or consumer of the 3-hydroxyacyl-CoA precursors or products), is affected by the [acetyl-CoA]/[CoA] and [NADH]/[NAD] ratios, with high ratios resulting in accumulation, whereas low ratios lead to oxidation of 3-hydroxyacyl-CoA. These results led them to conclude that PHAs accumulation is regulated by the [acetyl-CoA]/[CoA] and [NADH]/[NAD] ratios (Ren et al., 2009). Other allosteric regulatory effects exerted by key metabolites have been documented in other bacteria (Trotsenko and Belova, 2000).

Another example showing control at the level of enzyme activity was found in *Synechococcus* sp. In this cyanobacterium, PHB accumulation responds to nitrogen limitation, even in the presence of acetyl-CoA flux; PHB rapidly accumulates when transferred to nitrogen limiting conditions under light. This synthesis correlates with the appearance of PHB synthase activity in cell extracts, due to a post-translational activation of this enzyme mediated by acetyl phosphate. Because acetyl-phosphate is synthesized from acetyl-CoA through the activity of phosphotransacetylase, which is present only under nitrogen limitation, this enzyme acts as a switch indicating acetyl-CoA flux and acting as a signal of C:N balance (Miyake et al., 1997).

In *Rhodospirillum rubrum* PHB degradation is controlled at the level of enzymatic activity by an activator compound present in the cells. The activator compound was identified as a granule associated protein (phasin) named ApdA (activator of polymer degradation), that interacts with the surface of the granule and the PHA depolymerase, activating PHB degradation (Handrick et al., 2004).

A different mechanism for the regulation of PHAs metabolism, also through the control of enzyme activity, was reported in *R*. *eutropha* by Juengert et al. (2018). The constitutive presence of PHB synthase and PHB depolymerase during the different stages of growth led these authors to hypothesize that in order to avoid a futile cycle, their activities should be somehow regulated. Because covalent modification of enzymes by phosphorylation is a known way to modify their activities, they looked for phosphorylated residues in the PhaC1 synthase and the PHB depolymerase PhaZa1 during PHB accumulation and PHB degradation. Several phosphosites were identified in both enzymes, and phosphorylation of PhbC was dependent on the growth phase. Mutagenesis of the phosphorylatable residues showed that these modifications can affect their activities, so phosphorylation of these enzymes could also be part of the regulation of PHAs metabolism.

### Participation of Global Regulators in the Control of PHAs Metabolism

Coordination between PHAs metabolism with the rest of bacterial metabolism involves some global regulators. Examples have been described in diverse bacteria, and in some cases, interesting additional physiological changes have been reported to occur when altering these regulators. A brief summary of these regulators follows. The effects of modifications of these regulatory systems on PHA accumulation are summarized in Table 1.

**TABLE 1:** Regulatory systems controlling PHAs metabolism in diverse bacteria and the effect of their modification.

**Type of regulator**	**Bacterial species**	**Regulatory element**	**Effect on PHAs production/accumulation**	**Growth conditions**	**References**
Alternative sigma factors	*R*. *eutropha* H16 *P*. *putida* KT2440	RpoS	Deletion of *rpoS* increased PHAs mobilization during stationary phase.	Shake flasks. *R*. *eutropha* H16: Minimal medium, N-replete, no C source (PHB utilization medium). *P*. *putida* KT2440: Minimal medium, sodium octanoate.	Raiger-Iustman and Ruiz, 2008; Brigham et al., 2012
	*P*. *aeruginosa* PA14 and PAO1 *A*. *vinelandii* UW *P*. *chlororaphis* PA23	RpoS	Inactivation of *rpoS* had a negative effect on PHAs accumulation.	Shake flaks. *P*. *aeruginosa* PA14 and PAO1: Minimal medium, fructose. *A*. *vinelandii* UW: Minimal medium, N-limiting, sucrose. *P*. *chlororaphis* PA23: Minimal medium N-replete, glucose or octanoic acid.	Choi et al., 2011; Hernández-Eligio et al., 2011; Sharma et al., 2018
	*P*. *putida* KT2440	RpoN	Inactivation of *rpoN* increased PHAs accumulation 40% on octanoate but not gluconate.	Shake flasks. Minimal medium, N-limiting, octanoate or oleate.	Hoffmann and Rehm, 2004; Mozejko-Ciesielska et al., 2017
	*P*. *aeruginosa* PAK-N1	RpoN	Inactivation of *rpoN* abrogated PHAs synthesis on all conditions tested.	Shake flasks. Minimal medium, N-limiting or N-replete, gluconate or octanoate.	Hoffmann and Rehm, 2004
	*Synechocystis* sp. PCC 6803	SigE	Overexpression of *sigE* increased 2.3 times PHB production.	Minimal medium, N-limiting, glucose.	Osanai et al., 2013
Two-component system	*Synechocystis* sp. PCC 6803	Rre37	Overexpression of *rre*37 doubled PHB production.	Minimal medium, N-limiting, glucose.	Osanai et al., 2014
	*A*. *brasilense* SP7 *H*. *seropedicae* SmR1 *P*. *denitrificans* PD1222	NtrB NtrC	The *ntrB, ntrC* and/or *ntrBC* mutants produced higher amounts of PHB continuously during growth on N-replete conditions.	Batch fermentation or shake flasks. *A*. *brasilense* SP7: minimal medium, different concentrations of N, malate as C source. *H*. *seropedicae* SmR1: minimal medium, different concentrations of N on malate, glucose, fructose or xylose. *P*. *denitrificans* PD1222: mineral-salt medium, N-replete, succinate.	Sun et al., 2000; Sacomboio et al., 2017; Olaya-Abril et al., 2018
	*P*. *putida* CA-3 *P*. *putida* KT2442 *P*. *chlororaphis* PA23 *A*. *vinelandii* ATCC 9046	GacS	Disruption of the GacS sensor kinase had a negative effect on PHAs accumulation.	*P*. *putida* CA-3 and KT2442: PHAs not quantified. *P*. *chlororaphis* PA23: Mineral salt medium, N-replete, glucose or octanoate. *A*. *vinelandii* UW: mineral-salt medium, N-limiting with sucrose.	Castañeda et al., 2000; Ryan et al., 2013; Prieto et al., 2016; Sharma et al., 2018
Small RNAs	*S*. *meliloti* 2011	MmgR	Inactivation of *mmgR* increased PHB production 20% during stationary phase.	Mineral salt medium, N-limiting and surplus of sucrose.	Lagares et al., 2017.
	*A*. *vinelandii* UW136	RsmA	Inactivation of *rsmA* increased 25% PHB accumulation.	Shake flasks. Mineral-salt medium, N fixing conditions on sucrose.	Hernández-Eligio et al., 2012.
	*A*. *vinelandii*UW136	ArrF	Disruption of *arrF* gene reduced accumulation of PHB 75%.	Shake flasks. Mineral-salt medium, N fixing conditions on sucrose.	Muriel-Millán et al., 2014.
	*A*. *vinelandii* KCTC 23243	ArrF	Deletion of *arrF* gene increased accumulation of PHB 300-fold.	Shake flasks. Mineral-salt medium, N-replete on sucrose.	Pyla et al., 2009.
Stringent response	*R*. *etli* CE3	Rsh	Bacteroids with *rsh* gene inactivated were unable to accumulate PHB.	In symbiosis with *Phaseolus vulgaris*.	Calderón-Flores et al., 2005.
	*R*. *eutropha* H16	SpoT1 SpoT2	Stains unable to synthesize (p)ppGpp accumulated minor amounts of PHB, whereas increasing (p)ppGpp levels caused a 40% higher PHB accumulation.	Shake flasks. Nutrient broth with sodium gluconate.	Juengert et al., 2017.
	*P*. *putida* KT2440	RelA SpoT	A *relA*/*spoT* mutant accumulated mcl-PHAs in both optimal and nitrogen limiting conditions.	Shake flasks. Mineral-salt medium, N-replete and N-limiting, oleate.	Mozejko-Ciesielska et al., 2017
	*P*. *chlororaphis* PA23	RelA SpoT	Inactivation of *relA*/*spoT* decreases PHAs accumulation and changes monomeric composition of PHAs.	Mineral salt medium, N-replete, glucose or octanoate.	Sharma et al., 2018
Quorum sensing	*R*. *sphaeroides* 2.4.1	CerR CerI	Inactivation of *cerR/cerI* QS circuit accumulates a 2-fold higher content of PHB throughout aerobic growth.	Shake flasks. Mineral salt medium, succinate.	Kho et al., 2003
	*P*. *chlororaphis* PA23	AHL-deficient strain	Disruption of QS regulatory circuit decreases PHAs accumulation and changes the monomer composition of PHAs synthesized.	Shake flasks. Mineral salt medium, glucose or octanoate.	Mohanan et al., 2019
Oxygen-sensitive regulation	*P*. *extremaustralis* DSM 17835 *P*. *chlororaphis* PA23	Anr	Inactivation of *anr* decreases PHB accumulation under aerobic and microaerobic conditions.	Batch cultures or shake flasks. *P*. *extremaustralis* DSM 17835: Mineral-salt medium, octanoate and nitrate, with or without casein amino acids (CAS). *P*. *chlororaphis* PA23: Mineral-salt medium, glucose or octanoate.	Tribelli et al., 2010; Mohanan et al., 2019
	*H*. *seropedicae* SmR1	Fnr	A triple Fnr mutant showed a 50% reduction on PHB accumulation under low oxygen tension.	Shake flasks. NFbHPN-Malate mineral-salt medium, N-replete.	Batista et al., 2018
	*A*. *vinelandii* UW136	CydR	Inactivation of *cydR* increases accumulation of PHB during exponential growth phase.	Shake flasks. Mineral-salt medium, N-replete, glucose.	Wu et al., 2001
Phosphotransferase system	*A*. *vinelandii* UW136 *P*. *putida* KT2440	PTS^Ntr^	Mutations producing a non-phosphorylated form of EIIA^Ntr^, reduced PHAs accumulation. Inactivation of *ptsN* increased PHAs accumulation.	Shake flasks. *A*. *vinelandii* UW136: Peptone-yeast extract sucrose medium or mineral-salt medium, nitrogen fixing, sucrose. *P*. *putida* KT2440: Minimal SALT medium, octanoate.	Segura and Espín, 1998; Velázquez et al., 2007; Noguez et al., 2008
	*R*. *eutropha* H16	PEP-PTS PTS^Ntr^	Absence of EI and/or HPr decreased PHB content. Inactivation of *ptsN* increased PHB accumulation.	Batch cultures or shake flasks. Mineral-salts medium N-replete, gluconate or glycerol.	Pries et al., 1991; Kaddor and Steinbüchel, 2011; Kaddor et al., 2012
Other transcriptional regulators	*P*. *putida* KT2442	Crc	*crc* inactivation increased two times PHAs accumulation during exponential growth. Volumetric production increased 65%.	N-replete conditions. LB medium, octanoate as extra-carbon source.	La Rosa et al., 2014
	*P*. *putida* KT2442	PsrA	PsrA inactivation reduced PHAs accumulation 16 to 54%. More active β-oxidation. Higher content of shorter chain length monomers.	Mineral salt medium, N-limiting, octanoate, decanoate, glucose, fructose and succinate.	Fonseca et al., 2014
Direct PHAs-regulators	*Pseudomonas* sp. 61-3 *A*. *vinelandii*UW136	PhbR^a^	PhbR directly regulates *phbBAC* operon. Inactivation of *phbR* diminished PHB production. Overexpression of *phbR* in *Pseudomonas* sp. 61-3 enhanced PHB biosynthesis.	Shake flasks. *Pseudomonas* sp. 61-3: mineral-salt medium, N-limited, gluconate, octanoate, dodecanoate, or tetradecanoate. *A*. *vinelandii* UW136: Shake flasks. Mineral-salt medium nitrogen fixing, sucrose.	Matsusaki et al., 1998; Segura et al., 2000
	*P*. *putida* GPo1 *P*. *putida* U *P*. *putida* KT2442	PhaD	PhaD directly regulates *phaC* and *phaIF* operons. Inactivation of *phaD* reduced PHAs production and affected the number and the size of PHAs granules.	Shake flasks. NE2 mineral salt medium, octanoate. M63 mineral salt medium, N-limited, octanoate.	Klinke et al., 2000; Sandoval et al., 2007; de Eugenio et al., 2010b
Granule-associated regulators	*P*. *denitrificans* ATCC 17741 *R*. *eutropha* H16 *S*. *meliloti R*. *etli* CE3 *B*. *diazoefficiens R*. *sphaeroides* FJ1	PhaR (AniA) (rhizobia species)	PhaR is not a direct PHAs-synthesis regulator, although in *R*. *etli*, mutations in this gene decreased ≈40% the PHAs synthesis.	*R*. *etli* CE3: Shake flasks. Mineral salt medium, pyruvate.	Maehara et al., 1999; Povolo and Casella, 2000; York et al., 2002; Chou et al., 2009; Quelas et al., 2016
	*P*. *putida* KT2442	PhaF^b^	PhaF is not a direct PHAs-synthesis regulator, binds DNA in a non-specific manner. Involved in segregation of granules between daughter cells during cell division.	Shake flasks. M63 mineral salt medium N-limited, octanoate.	Galán et al., 2011

*^*a*^: PhbR is a member of the AraC/XylS family, not related to PhaR (AniA). ^*b*^: Although PhaF, like PhaR, is able to bind to DNA and granules, its amino acid sequence shows no similarity to PhaR.*

#### Alternative Sigma Factors in the Control of PHAs Metabolism

For transcriptional regulation, the selectivity of RNA polymerase is controlled by interaction with two types of regulatory proteins: sigma factors and transcription factors (Ishihama, 2012). The utilization of sigma factors alternative to the housekeeping σ70 provides a mechanism for bacterial responses to many stresses, by redirecting transcription initiation to the simultaneous regulation of large numbers of genes. Among these alternative sigma factors, RpoS increases under conditions of nutrient stress, or during the stationary phase, leading to general stress resistance (Battesti et al., 2011). PHB accumulation and mobilization are related to the nutrient status of bacteria (López et al., 2015), so RpoS has a role in the regulation of PHAs metabolism in some bacteria (Brigham et al., 2012).

In *R*. *eutropha*, inactivation of *rpoS* induced no change during PHB production, however, it exhibited an increased PHB depolymerization when polymer utilization was induced, in comparison to the wild-type strain H16. Therefore, RpoS was proposed to have a role in PHB utilization (Brigham et al., 2012).

A similar role was found in *P*. *putida* (Raiger-Iustman and Ruiz, 2008). In this organism inactivation of *rpoS* had no effect in PHAs accumulation, however, when entering the stationary phase, the polymer content of the mutant diminished faster than in the wild type, resulting in a lower PHAs accumulation. The transcript containing *phaC*1 and *phaZ* (coding for one of the two PHA synthases present in this bacterium and the PHA depolymerase) was overexpressed in the stationary phase, probably causing a higher depolymerization activity. The authors concluded that RpoS participates in the negative regulation of the promoter of *phaC*1*-phaZ*, probably in an indirect manner and suggested that PhaD, a transcriptional regulator of the TetR family, could be an intermediary.

In *Pseudomonas aeruginosa*, Choi et al. (2011) reported that *rpoS* inactivation in PA14 and PAO1 strains showed a strong negative effect on PHAs accumulation when grown on fructose, however, on decanoic acid, only the PA14 mutant showed a similar negative effect. These results suggest RpoS sigma factor participates in the transcription of some PHAs biosynthetic genes with differences depending on the strain. It is interesting to note that, besides the observed effect on the amount of polymer accumulated, inactivation of *rpoS* shifted the monomeric composition of the PHAs to longer hydroxyalkanoates (Choi et al., 2011). PHAs depolymerization has also been linked to *rpoS* expression and to tolerance to oxidative and thermal stresses, through an unknown mechanism (Ruiz et al., 2004). This could be related to the reported need of PHB utilization for ATP and ppGpp synthesis (Ruiz et al., 2001), or as part of the role that PHAs metabolism has in stress resistance in several bacteria (López et al., 2015).

The role of sigma factor RpoS has also been elucidated in *A*. *vinelandii* UW136. In this bacterium, the synthesis of PHB occurs mainly during the stationary growth phase. Accordingly, RpoS was shown to participate in the transcription of the PHB biosynthetic operon *phbBAC* and of *phbR*, which codes for the transcriptional activator of the *phb* genes (Hernández-Eligio et al., 2011).

In *Pseudomonas chlororaphis* PA23, inactivation of *rpoS* negatively affected the amount of PHAs, due to down-regulation of *phaC*1, *phaC*2, and the two phasin genes *phaF* and *phaI* (Sharma et al., 2018).

Because the alternative sigma factor RpoN (sigma 54) is involved in the regulation of nitrogen metabolism and nitrogen limitation induces PHAs biosynthesis, the participation of this sigma factor on the accumulation of these polymers was evaluated in *P*. *putida* KT2440 and in *P*. *aeruginosa* PAK by Hoffmann and Rehm (2004, 2005). They studied PHAs accumulation in the wild type and *rpoN*-negative mutant strains of these bacteria under nitrogen excess or nitrogen limitation. In *P*. *aeruginosa*, inactivation of *rpoN* abrogated PHAs accumulation on gluconate and octanoate, suggesting that RpoN participates in the expression of a gene involved in PHAs metabolism. The expression of transacylase PhaG (now reclassified as a 3-hydroxyacyl-ACP tioesterase), needed for the biosynthesis of PHAs from carbohydrates, was found to be inducible under nitrogen limitation in both species and was dependent on RpoN in *P*. *aeruginosa*, but not in *P*. *putida* (Hoffmann and Rehm, 2004). It was also suggested that RpoN is a negative regulator of *phaF*, which was thought to be a negative regulator of *phaC*1 (Hoffmann and Rehm, 2005). In the case of *P*. *putida* KT2440 it is interesting to note that, although *rpoN* inactivation had no effect on PHAs accumulation on gluconate, it increased their accumulation 40% on octanoate, reaching the highest accumulation in these experiments (Hoffmann and Rehm, 2004). A similar result was obtained by Mozejko-Ciesielska et al. (2017) while analyzing the effect of *rpoN* inactivation in the same *P*. *putida* strain. They also found a significant increase in the accumulation of mcl-PHAs in the *rpoN* mutant when growing on octanoate under nitrogen limitation. Therefore, the inactivation of *rpoN* could be further studied as a candidate for strain improvement in *P*. *putida*, which is the model bacterium for mcl-PHA synthesis.

Another example illustrating the participation of an alternative sigma factor on the control of PHB metabolism is found in the non-nitrogen-fixing cyanobacterium *Synechocystis* sp. PCC 6803 (Osanai et al., 2013). In this microorganism, PHB accumulation increases under phosphorous or nitrogen starvation. Transcription levels of PHB biosynthetic genes increase after nitrogen depletion. Coincidentally, the expression of the sigma factor named SigE also increased under this condition. A strain overexpressing *sigE* showed higher mRNA levels of the *phb* biosynthetic genes, with respect to those of the wild type, during the nitrogen-replete stage. This overexpression of *sigE* also increased the levels of the PHB biosynthetic enzymes during nitrogen limitation. This strategy increased 2.4 times the accumulation of PHB (Osanai et al., 2013).

Also in *Synechocystis* sp. PCC 6803, the response regulator Rre37 of a two-component system, has been shown to participate in the control of PHB accumulation (Osanai et al., 2014). This protein plays a role in carbon metabolism because the overexpression of *rre*37 up-regulated genes related to sugar catabolism, under both nitrogen-replete and nitrogen-depleted conditions and decreased glycogen accumulation. The expression of the *phb* biosynthetic genes was also up-regulated by Rre37, mainly under nitrogen depletion, and almost doubled PHB accumulation. Interestingly, overexpression of both Rre37 and SigE further increased PHB accumulation to 2.9 times. The Mr and monomer composition of PHB were not altered by the double overexpression of SigE and Rre37. Thus, Rre37 is a regulatory protein that changes the metabolic flow from glycogen to polyhydroxybutyrate and is probably participating in the same regulatory circuit with SigE, through an unknown mechanism. Besides suggesting the participation of sigma factor SigE and Rre37 in the control of transcription of the *phb* biosynthetic genes, these are good examples of how changing the regulatory networks affecting PHB metabolism can be a powerful approach, because not only the genes participating in PHB accumulation altered their expression when overexpressing *sigE* or *rre*37, but a whole set of genes related with carbon metabolism (glycogen catabolism), among other changes, so PHB production improvement was due to a higher expression of the *phb* genes, but also to a change in carbon distribution from glycogen to PHB (Osanai et al., 2013, 2014). It is noteworthy that increasing the enzymatic activities involved in the synthesis of PHAs by introducing the biosynthetic genes of *R*. *eutropha* into *Synechocystis* 6803 did not really enhance PHB accumulation, suggesting this is not the only rate-limiting point for PHB synthesis (Sudesh et al., 2002).

PsrA, a positive regulator of *rpoS* expression that also represses the fatty acid β-oxidation operon *fadBA*5 in *P*. *aeruginosa* (Kojic and Venturi, 2001; Kang et al., 2008), has been implicated in the fatty acid - PHA metabolic network in *P*. *putida* KT2440. In this bacterium, inactivation of *psrA* reduced PHAs accumulation between 16 and 54%, depending on the carbon source. This effect was explained as a consequence of a more active β-oxidation pathway induced by the *psrA* inactivation, although direct regulation of *pha* genes by PsrA or changes in their expression caused by a negative effect on *rpoS* expression, were not ruled out. In fact, overexpression of *phaC*2 gene was observed in the *psrA* mutant. Interestingly, a change in the composition of the polymers was also observed in this mutant, with a higher content of short chain length monomers (Fonseca et al., 2014).

#### Control by the Stringent Response

Because PHAs in many organisms are produced under conditions of nutritional limitation, and adaptation to nutrient deprivation is mediated in many bacteria by the so-called stringent response (Boutte and Crosson, 2013), the participation of this stress response regulatory mechanism in the control of PHAs metabolism has been analyzed in several bacteria (López et al., 2015). Stringent response mediates adaptation to changes in nutrient availability by reprograming transcription of a high percentage of genes. This response is mediated by the nucleotides guanosine tetraphosphate (ppGpp) and guanosine pentaphosphate (pppGpp), the so-called alarmone, whose levels are controlled by the enzymes RelA (ppGpp synthetase), and SpoT (ppGpp synthetase/hydrolase) (Boutte and Crosson, 2013).

The first report showing a possible connection between stringent response and PHAs metabolism was found in *P*. *aeruginosa* GPo1 through the analysis of a *phbZ* minus mutant (Ruiz et al., 2001). It was shown that utilization of the PHB reserve is needed for the proper synthesis of ppGpp, and this, in turn, has an effect on stress resistance to ethanol and high temperature, however, no regulatory relationships between stringent response and PHAs metabolism were demonstrated.

Later, the analysis of a *Rhizobium etli* mutant unable to accumulate (p)ppGpp, showed that it was affected in amino acids and nitrate utilization, nodulation, nitrogen fixation and also was unable to accumulate PHB (Calderón-Flores et al., 2005).

In *R*. *eutropha*, the role of stringent response was investigated by inactivating the (p)ppGpp synthetase/hydrolase and (p)ppGpp synthetase genes of this bacterium. Double mutants, unable to synthesize (p)ppGpp, accumulated a minor amount of PHB, whereas unusually high levels of this polymer were obtained in a strain with increased levels of (p)ppGpp, generated by overexpression of the (p)ppGpp synthase SpoT2 in the absence of (p)ppGpp hydrolase. The artificial induction of stringent response through the addition of the amino acid analogs norvaline or serine hydroxamate, increased the contents of PHB during the stationary phase. Therefore, a positive correlation between the amount of ppGpp and the accumulation of PHB was suggested. The composition of the polymer was not affected by the induction of the stringent response. The authors concluded that the concentrations of (p)ppGpp affect PHB accumulation by negatively regulating PHB depolymerization, and suggested this could be done through allosteric regulation and covalent modification of the enzymes involved. They identified the main PHB depolymerase PhaZa1 as the target for this control (Juengert et al., 2017).

In *P*. *putida* KT2440 accumulation of PHAs is also induced under nitrogen limiting conditions. Interestingly, disruption of strict response by the inactivation of *relA* and *spoT* genes eliminated the need for nitrogen limitation, so the polymers were accumulated to the same level under both conditions. The *relA/spoT* mutant was able to accumulate 2.8 times the PHAs produced by the wild-type strain under no nitrogen limitation (Mozejko-Ciesielska et al., 2017). However, lower accumulation of PHAs was observed under nitrogen limitation, in comparison to the wild-type strain. In this case, the expression of the PHA depolymerase gene (*phaZ*) was lower in the absence of (p)ppGpp and could explain in part the phenotype observed. This altered regulation could be further investigated in order to promote PHAs accumulation under culture conditions allowing growth to high cell densities, with no need for a change to nutritional limitation to start producing the polymers.

More recently, a report in *P*. *chlororaphis* PA23, a bacterium used for biocontrol, showed that mutations in the regulatory genes *relA/spoT* significantly decreased PHAs accumulation, and also resulted in altered PHAs gene expression. In this study, it was shown that the two PHA synthase genes *phaC*1, *phaC*2, and the two phasin genes *phaF*, and *phaI* were also significantly down-regulated in *gacS* and *rpoS* mutants, reducing the amount of PHAs (Sharma et al., 2018). Interestingly, this work illustrates that the modification of stringent response and of other regulatory circuits can also change the monomeric composition of PHAs. For the three regulatory mutants (*relA/spoT, gacS*, *rpoS*), the PHAs produced on glucose contained significantly higher amounts of 3-hydroxyhexanoate and/or 3-hydroxyoctanoate, and the content of 3-hydroxydecanoate was lower, as compared to the parental strain. For the PHAs synthesized on octanoate, only in the case of the *gacS* mutant the monomer composition changed (Sharma et al., 2018).

#### Regulation by Quorum Sensing

Bacterial quorum sensing (QS) is a communication process that involves self-produced extracellular signaling molecules, called autoinducers, which can accumulate into levels that are required to collectively alter global patterns of gene expression (Papenfort and Bassler, 2016). The bioluminescent marine bacterium *Vibrio harveyi* is a good example of a canonical QS circuit, with a LuxR-type receptor that detects autoinducers. This bacterium is capable to synthesize PHB only at a high cell density and this production is positively controlled by the LuxR-autoinducers circuit (Sun et al., 1994; Miyamoto et al., 1998). Although the exact molecular mechanism is unknown, it is proposed that *V*. *harveyi* autoinducer leads to the increased expression of *luxR*; the LuxR activator in turn stimulates at high cell density the expression of PHB synthesis and luminescence (Miyamoto et al., 1998). Similarly, the physiological implication of PHB synthesis in relation to the luminescence is not clear, but it is possible that the polymer in *V*. *harveyi* serves as an energy source for maintaining cell viability in the stationary growth phase.

Interestingly, it has been found that the PHB synthesis of *Rhodobacter sphaeroides*, a facultative photosynthetic bacterium, is controlled in a manner analogous to the induction of cell luminescence. However, unlike the positive regulation of the PHB synthesis of *V*. *harveyi*, the PHB synthesis of *R*. *sphaeroides* is under negative regulation. In this bacterium, the *cerR/cerI* QS circuit codes for LuxR-type regulator and the enzyme that synthesize the autoinducer molecule, respectively (Ye et al., 2013). Under aerobic growth conditions, mutations in the *cerR/cerI* genes resulted in a 2-fold increase in the cellular PHB content, with respect to the wild type, and this increase is coincidental with a proportional increase of the PHB synthase enzymatic activity. Moreover, the transcriptional levels of *phaC* gene were also 2-fold higher than the corresponding values of the wild type (Kho et al., 2003). The potential of mutations in *cerR/cerI* genes has not been fully explored.

Another example illustrating the participation of QS in the control of PHA gene expression is found in *P*. *chlororaphis* PA23. There are three distinct QS regulatory circuits in this bacterium, PhzRI, CsaRI, and AurRI, but only the Phz system has been characterized. An AHL-deficient strain (comparable to a triple *phzI*/*csaI*/*aurI* QS mutant) resembles stringent response-defective *P*. *chlororaphis* PA23: it shows a significantly decreased PHAs accumulation, a radical change in PHAs composition, and down-regulation of *phaC*1, *phaZ*, *phaC*2, *phaD*, *phaF*, and *phaI*. The search for regulatory sequences in these genes revealed the presence of a region with 15/20 conserved nucleotides upstream of *phaZ* and *phzF*, named “*phz*-box”; nevertheless, direct QS regulation of *phaZ* and *phaF* has not been demonstrated (Mohanan et al., 2019).

#### Control by Oxygen Responsive Regulators

In addition to the previously described allosteric regulatory mechanism controlling PHB synthesis in response to oxygen limitation in *R*. *eutropha* (Oeding and Schlegel, 1973) and *A*. *beijerinckii* (Senior and Dawes, 1973), other mechanisms for the control by oxygen have been described, but operate at the level of transcription.

In *Pseudomonas extremaustralis* PHB production is induced under micro aerobic and anaerobic cultures. The redox transcriptional regulator Anr (Fnr-like), which regulates several genes during the transition from aerobic to anaerobic growth, is involved in this control. The Anr protein contains a [4Fe-4S]^+2^ cluster that suffers oxidation, changing its DNA binding affinity, thus sensing the redox state of the cell. Inactivation of *anr* decreased expression of *phaC* and *phaR* (coding for the transcriptional regulator of *pha* biosynthetic genes) and PHB production under aerobic and micro aerobic conditions. The presence of two putative binding sites in the regulatory region of these genes suggested a direct regulation of transcription of these genes by Anr (Tribelli et al., 2010).

In *Herbaspirillum seropedicae* SmR1, a nitrogen-fixing endophyte, where PHB has an important role in the maintenance of the intracellular redox balance, it was found that two of the three Fnr regulators encoded in this bacterium control expression of the *phaC* synthase gene. A triple Fnr mutant showed a 50% reduction on PHB accumulation under low oxygen tension, with respect to the wild-type strain (Batista et al., 2018).

A role for Anr in the control of mcl-PHA metabolism has also been shown in *P*. *chlororaphis* PA23. Inactivation of *anr* significantly reduced PHAs accumulation, although their monomeric composition was not drastically changed. Although expression of *phaC*1, *phaZ*, *phaC*2, *phaD*, *phaF*, and *phaI* genes related to PHAs metabolism was analyzed, only *phaF* showed a significant reduction in the mutant; nevertheless, Anr-recognition sequences were not found upstream of *phaF* (Mohanan et al., 2019).

The participation of an Fnr homolog (CydR) in the control of PHB metabolism in response to oxygen has also been reported in *A*. *vinelandii*, where this transcriptional regulator seems to be a repressor of the biosynthetic genes (Wu et al., 2001). This regulation is further described in section “Regulation of PHB Synthesis in *Azotobacter*.”

#### Control by Phosphate Limitation Through PhoB

In *Acinetobacter* sp. PHB accumulation is induced by phosphate starvation and a regulatory mechanism has been proposed. Schembri et al. (1995) showed that phosphate limitation induces transcription of the *pha* biosynthetic locus. The presence of two potential Pho-box consensus sequences, upstream of two transcription start points, and the requirement of PhoB (the *pho* regulon activator) and phosphate limitation for the expression of these transcripts, showed direct positive regulation of these genes by the transcriptional regulator PhoB under phosphate limitation.

#### The Carbon Catabolite Regulator Crc

Control by the global regulator Crc has been demonstrated in *P*. *putida* KT2442 (La Rosa et al., 2014). Crc is a protein that in *Pseudomonas* has a key role in carbon catabolite repression, a process that allows to first assimilate a preferred carbon source among a mixture of several compounds. Crc acts post-transcriptionally by binding to its target mRNAs on motifs close to their ribosome binding site, affecting translation (Rojo, 2010). In *P*. *putida*, La Rosa et al. (2014) showed that Crc links PHAs metabolism with the regulatory networks controlling carbon utilization. They identified a Crc binding motif on the PHA synthase gene *phaC*1 mRNA, and demonstrated that Crc inhibits *phaC*1 mRNA translation and not transcription. They also showed that the activity of this regulator is affected both by the carbon source present and by the C/N ratio of the medium. This is carried out by the antagonist small RNAs CrcZ and CrcY, which sequester Crc and the expression of these RNAs is higher under conditions in which PHAs synthesis is high and Crc is strongly antagonized. They also reported that Crc negatively controls PHAs synthesis when growing in media with a balanced carbon/nitrogen ratio. Under unbalanced carbon/nitrogen conditions (where PHAs synthesis is allowed), Crc had no activity. Interestingly, in the *P*. *putida crc* negative mutant, the requirement for nitrogen limitation for polymer synthesis was lost, allowing to accumulate the same percentage of PHAs under nitrogen replete conditions. In LB medium with octanoate, the highest accumulation percentages and also the highest PHAs volumetric production were obtained with the *crc* mutant, eliminating the need to restrict growth by limiting nitrogen (La Rosa et al., 2014). Therefore, this is another example of the potential benefits of modifying the regulatory systems controlling PHAs metabolism for strain improvement.

#### The Two-Component System NtrB-NtrC

NtrB and NtrC proteins are the sensor kinase (NRII) and the response regulator (NRI) of a two-component regulatory system that in diverse bacteria is a key player in the control of expression of many genes of nitrogen metabolism (van Heeswijk et al., 2013). These regulators are also involved in the regulation of PHB in some bacteria.

In *Azospirillum brasilense* SP7, PHB production is inhibited by the presence of high ammonia concentration. Inactivation of *ntrB* or *ntrC* considerably increased PHB accumulation (up to 9-fold) on different C/N ratios. The *ntrB* or *ntrC* mutants couple PHB production and growth, producing PHB both during the growth phase and stationary phase. The absence of these regulators eliminates the inhibitory effect of ammonia, and interestingly, greatly diminished the respiration of *A*. *brasilense* (Sun et al., 2000). All these phenotypes show that NtrB and NtrC somehow regulate PHB metabolism; therefore, the corresponding mutants are interesting starting strains for future improvements.

In *H*. *seropedicae* SmR1, an *ntrC* mutant was also shown to accumulate higher levels of PHB than the wild type (up to 2-fold increase). The inactivation was found to increase the activity of glucose-6-phosphate dehydrogenase (a reaction producing NADPH), leading to a 2.1-fold increase in the NADPH/NADP + ratio. This increase in NADPH was proposed to cause the increased synthesis of the polymer (Sacomboio et al., 2017).

The NtrB regulator of *Paracoccus denitrificans* PD1222 was also shown to somehow regulate PHB accumulation. In this bacterium inactivation of *ntrB* induced the overproduction of the polymer (5-fold), when growing on nitrate, and also increased expression of the PHA synthase gene *phaC*. An interesting metabolic change was also observed. The acetyl-CoA concentration increased 4-fold in the *ntrB* mutant. Therefore, the increased accumulation of PHB in this mutant was a combination of the effect on *phb* gene expression with the induction of a metabolic change caused by altering the regulatory network (Olaya-Abril et al., 2018).

#### The Two-Component System GacS-GacA

Many other transcriptional regulatory systems have been reported to participate in the control of PHAs metabolism. One very interesting is the GacS-GacA two-component system, where GacS functions as a sensor histidine kinase protein that phosphorylates GacA, which is the response regulator that when phosphorylated activates transcription of its target genes. The GacS-GacA system is conserved in gram-negative bacteria. Its participation in the control of PHB metabolism was discovered in *A*. *vinelandii* while looking for regulators of alginate synthesis, because inactivation of *gacS* had a negative effect on the synthesis of this polysaccharide, but also on PHB accumulation (Castañeda et al., 2000). It was shown later that phosphorylated GacA is a positive regulator of PHB synthesis, *rpoS* expression (Castañeda et al., 2001), and for the expression of the small regulatory RNAs RsmZ/Y, that interact with the translational regulatory protein RsmA (Manzo et al., 2011). This regulation is further described in section “Regulation of PHB Synthesis in *Azotobacter*.”

In *P*. *putida* CA-3 the participation of the GacS-GacA on the control of mcl-PHAs metabolism was found by random mini-Tn5 mutagenesis. A mutant unable to accumulate PHAs had disrupted the *gacS* gene. Transcription of the regulatory small RNAs RsmY and RsmZ, known to be regulated by GacS-GacA in other *Pseudomonas*, was not affected in the *gacS* mutant. Transcription of the PHA synthase *phaC*1 was also unaffected, but evidence of a lower content of the synthase enzyme suggested a post-transcriptional control (Ryan et al., 2013). In *P*. *putida* KT2442, inactivation of the sensor kinase GacS also reduced PHAs accumulation, but in this case, a reduction in the transcription rate of the PHAs gene cluster was observed and this was restored by expressing PhaD, an activator of the *pha* genes discussed in section “PhaD” (Prieto et al., 2016).

As previously mentioned, in *P*. *chlororaphis* PA23 inactivation of *gacS* strongly decreased PHAs accumulation and this correlated with a considerably diminished expression of genes *phaC*1, *phaC*2, *phaD, phaF, phaI* and *phaZ*. Interestingly, the *gacS* mutation also changed the monomeric composition of the mcl-PHAs produced by this bacterium (Sharma et al., 2018).

#### Regulatory Small RNAs

Small non-coding RNAs (sRNAs) play important regulatory roles in bacteria. Many of them act as antisense RNAs on multiple target mRNAs, other sRNAs act by binding to proteins, some of them regulatory, affecting their activity (Gottesman and Storz, 2011).

In the regulation of PHB metabolism, some examples of regulation by small RNAs have been documented. Free-living *Sinorhizobium meliloti* cells accumulate PHB under nitrogen-limiting conditions. For that reason, Lagares et al. (2017) studied a small RNA called MmgR (Makes more granules Regulator), whose expression is regulated by the amount of nitrogen source. They found that MmgR negatively regulates PHB accumulation under conditions of N starvation and C surplus. Inactivation of *mmgR* gene increased PHB production by 20% with respect to the wild type, without affecting growth. The increase in PHB was even higher when using higher C/N ratios. The overproduction occurred during the stationary phase, but not during the exponential growth, so PHB production was still affected by nutrient limitation. The *mmgR* mutation increased the number of granules and also resulted in the overexpression of two phasin proteins. So, MmgR sRNA participates in the regulation of PHB accumulation in this bacterium, through an unknown mechanism. This mutation is another candidate for further studying its potential to improve PHB production.

A regulatory small RNA called ArrF has been implicated in the regulation of PHB accumulation in response to iron in *A*. *vinelandii* (Pyla et al., 2009; Muriel-Millán et al., 2014). Iron-limiting conditions increase PHB accumulation in this bacterium (Page, 1982). The ArrF RNA participates in this regulation by affecting translation of its target, the mRNA of *phbR*, through complementary binding, although the effects of this interaction probably differ between strains (Pyla et al., 2009; Muriel-Millán et al., 2014). This is further described in section “Regulation of PHB Synthesis in *Azotobacter*.”

In *Pseudomonadaceae*, the non-coding small RNAs called RsmZ/Y/X regulate secondary metabolism and carbon storage (Lapouge et al., 2008). These RNAs, together with the RsmA protein, constitute a system of post-transcriptional regulation where RsmA acts as a translational repressor by binding to its mRNA targets, and the small RNAs counteract this repression through binding to RsmA. In *A*. *vinelandii*, inactivation of *rsmA* results in increased PHB production, because RsmA represses translation of the PHB biosynthetic operon *phbBAC* and of the *phbR* gene that codes for its transcriptional activator. At least two of the eight small RNAs present in this bacterium (RmZ1 and RsmZ2) have been shown to specifically bind RsmA and counteract this repression (Manzo et al., 2011; Hernández-Eligio et al., 2012). This regulation and its connection with the GacS/GacA two-component system will be described in section” Regulation of PHB Synthesis in *Azotobacter*.”

#### The PTS^Ntr^ Regulatory System

Another regulatory mechanism controlling the synthesis of PHB is carried out by the nitrogen-related phosphotransferase system (PTS^Ntr^). The participation of this system in the control of PHB synthesis was first reported in *A*. *vinelandii* (Segura and Espín, 1998). This system is homologous to the carbohydrate PTS used in many bacteria for uptake and phosphorylation of different sugar substrates. PTS^Ntr^ is present in most gram-negative bacteria and is composed of EI^Ntr^, Npr and EIIA^Ntr^ proteins encoded by the *ptsP, ptsO* and *ptsN* genes, respectively. These proteins participate in a phosphorylation cascade where EIIA^Ntr^ seems to be the final phospho-acceptor. The PTS^Ntr^ seems to be exclusively involved in regulatory functions (Pflüger-Grau and Görke, 2010). In *P*. *putida*, inactivation of PTS^Ntr^ components reduced the synthesis of PHAs by increasing the level of unphosphorylated EIIA^Ntr^ (Velázquez et al., 2007). In a similar way, *A*. *vinelandii* mutants where EIIA^Ntr^ is present in an unphosphorylated form are unable to produce PHB (Segura and Espín, 1998; Noguez et al., 2008). The mechanism by which the PTS^Ntr^ system regulates the synthesis PHB has been widely investigated in *A*. *vinelandii* and is described in section “Regulation of PHB Synthesis in *Azotobacter*.”

An interesting connection between PTS^Ntr^ and the stringent response was found in *R*. *eutropha* (Karstens et al., 2014). In an attempt to clarify the role of PTS^Ntr^ in the control of PHB metabolism in this bacterium, these authors screened for proteins able to interact with EIIA^Ntr^. The bifunctional ppGpp synthase/hydrolase SpoT1 was shown to interact, but only with the unphosphorylated form of EIIA^Ntr^. This could, in turn, alter the ppGpp levels, thus affecting PHB formation and the expression of genes responsive to this alarmone. A role for PTS^Ntr^ in the control of (p)ppGpp levels has also been described in *Caulobacter crescentus*, although its relation with PHAs metabolism was not investigated (Ronneau et al., 2016).

The participation in the control of PHB metabolism, not of the PTS^Ntr^ system, but of proteins of the sugar-specific-PTS system, has been documented in *R*. *eutropha* (Pries et al., 1991). Inactivation of *ptsH* or *ptsI* genes, coding for the components of the PTS, Enzyme I (EI) and the histidine phosphocarrier protein (HPr), respectively, caused a lower accumulation of PHB. These mutants showed a higher rate of PHB degradation, so the authors proposed that the PTS could be controlling, by phosphorylation, the activity of the PHB mobilizing enzymes or the affinity of a regulatory element controlling them (Pries et al., 1991). Later, Kaddor et al. (2012) reported a proteome analysis of a *ptsHI* double mutant of *R*. *eutropha* and showed that these mutations caused a down-regulation of the PHB biosynthetic acetoacetyl-CoA reductase and of two phasin proteins; also enzymes of central catabolism (Entner-Doudoroff pathway, the tricarboxylic acid cycle) were down-regulated in the mutant, whereas enzymes of gluconeogenesis showed enhanced expression in the mutant. Together, these changes explained the PHB-leaky phenotype observed.

It is interesting to point out that the permease components (EIIB and EIIC proteins/domains) that together with EI and Hpr proteins constitute a complete PTS for the transport of carbohydrates are not present in *R eutropha*, so EI and Hpr are likely to be involved in regulatory functions of carbon and PHB metabolism (Kaddor and Steinbüchel, 2011). Strikingly, this bacterium also lacks *ptsP* and *ptsO* genes of the PTS^Ntr^, but has a homologous to *ptsN* (IIA^Ntr^, H16_A0384) (Kaddor and Steinbüchel, 2011; Kaddor et al., 2012). Inactivation of *ptsN* increased PHB accumulation, as in *A vinelandii*, and *P*. *putida* (Kaddor and Steinbüchel, 2011), and phosphorylation of EIIA^Ntr^ through EI and Hpr has been shown and it favors PHB accumulation (Krausse et al., 2009).

### Specific Regulators of PHAs Metabolism

In different bacteria, some regulators specifically controlling the expression of *pha* genes, have been described. Here, we review the main transcriptional regulators reported.

#### PhbR

The genes encoding proteins involved in PHAs synthesis are often located in conserved PHAs gene clusters, depending if the bacteria are scl- or mcl-PHA producers. In some PHB-producing bacteria, like *A*. *vinelandii* (Peralta-Gil et al., 2002), *Azotobacter* FA8 (Pettinari et al., 2001), *Pseudomonas* sp. strain 61-3 (Matsusaki et al., 1998), and *Pseudomonas* sp. USM 4-55 (Tan et al., 2010), the *phbA*, *phbB* and *phbC* genes are clustered in the same *locus* and are organized in a single transcriptional unit. In all these cases, upstream of the *phbBAC* operon and in the opposite direction, the gene called *phbR* is located. This gene codes for a member of the AraC/XylS family of transcriptional regulators, with a typical DNA binding domain helix-turn-helix (HTH). It is important to note that Matsusaki et al. (1998) reported for the first time the similarity between PhbR and other proteins belonging to the AraC family, such as OruR of *P*. *aeruginosa* and the virulence-associated regulator of *Mycobacterium tuberculosis*; thus, the ORF was referred to as *phbR*, and it is not related to other regulators also called PhaR, that are discussed later. In *A*. *vinelandii* and *Pseudomonas* sp. strain 61-3 it was demonstrated that PHB biosynthesis is positively regulated by PhbR, which activates transcription of the *phbBAC* biosynthetic operon (Matsusaki et al., 1998; Peralta-Gil et al., 2002; Hernández-Eligio et al., 2011).

Although the majority of bacteria accumulate either scl-PHAs or mcl-PHAs, some of them, like *Pseudomonas* sp. strain 61-3, are able to synthesize and accumulate a blend of PHB homopolymer and a random P(3HB-co-3HA) copolymer. This is possible because these bacteria possess two types of PHAs biosynthetic gene loci. Interestingly, when the activator *phbR* was overexpressed *in trans*, *Pseudomonas* sp. strain 61-3 increased its polymer content (51%). Furthermore, the overexpression of *phbR* enhanced PHB biosynthesis in this bacterium, resulting in an increase of the polymer content and enrichment of the 3HB fraction in the whole polymer (94 mol%) (Matsusaki et al., 1998).

#### PhaD

Most *Pseudomonas* species belonging to the ribosomal RNA-homology group I synthesize mcl-PHAs, whose synthesis requires the proteins encoded by the *phaC*1*ZC*2*DFI* cluster. This cluster contains two polymerases (PhaC1 and PhaC2), a depolymerase (PhaZ), and three proteins (PhaDFI) involved in granule formation and in the regulation of gene expression. PhaD is a TetR-family transcriptional regulator that in *P*. *aeruginosa* GPo1 (formerly *Pseudomonas oleovorans* GPo1; Klinke et al., 2000; Ramos et al., 2005), *P*. *putida U* (Sandoval et al., 2007) and *P*. *putida* KT2440 (de Eugenio et al., 2010b), positively regulates PHAs synthesis. Inactivation of *phaD* reduces not only the production of the polymers, but also affects the size and number of the PHAs granules (Klinke et al., 2000). This regulation is performed in a direct manner, since PhaD binds to the promoter region of synthase *phaC*1*ZC*2*D* and *phaIF* operons, auto activating its expression and that of the other *pha* genes. This phenomenon is carbon source dependent, with higher activation on the preferred carbon source octanoate and this, together with 3D modeling of PhaD, allowed the authors to suggest the participation of a β-oxidation metabolite as an effector (de Eugenio et al., 2010b).

The finding that PhaD simultaneously activates expression of the PhaZ depolymerase and the PHA synthases is in agreement with the conclusion that synthesis and mobilization in *Pseudomonas* is a cyclic process allowing PHAs turnover to achieve a metabolic balance (de Eugenio et al., 2010a). However, knowledge about how this potentially futile cycle is regulated or balanced is relatively limited. As discussed in section “Control of Enzymatic Activity,” this could be accomplished through allosteric control of the enzymes involved. PHA synthase and the fatty acid oxidation complex, the provider or consumer of 3-hydroxyacyl-CoAs (precursors or depolymerization products), are affected by metabolites like R-3-hydroxyacyl-CoA, free CoA, acetyl-CoA, NADH and NAD; thus, the balance of this cycle could be determined by the metabolic status of the cell (Ren et al., 2009). In bacteria like *R*. *eutropha*, where a similar situation of constitutive *phb* gene expression and PHB turnover also occur (Doi et al., 1990; Brigham et al., 2012), the control of this cycle seems to be achieved also by controlling enzyme activities, through allosteric regulation by the intracellular levels of acetyl-CoA and free CoA and its redox state (Oeding and Schlegel, 1973; Haywood et al., 1988). As mentioned in sections “Control of Enzymatic Activity” and “Control by the Stringent Response,” the activities of the PHB synthase or PhaZa1 depolymerase can also be controlled by (p)ppGpp levels or through phosphorylation (Juengert et al., 2017, 2018).

#### Granule-Associated Regulators

When PHAs are synthesized, they accumulate as granules, which are composed of 97.5% PHA, 2% proteins, and some lipids (Jendrossek and Pfeiffer, 2014). Four types of proteins are found associated to the granules: structural proteins called phasins, PHA metabolism enzymes, like PHA synthases and depolymerases, and regulatory proteins (Pötter and Steinbüchel, 2005).

##### PhaR (PhaF, AniA)

The accumulation of PHAs granules in the cytoplasm of producing bacteria is strongly influenced by phasins, which are amphipathic low-molecular-weight proteins that influence the number and size of granules (Pötter and Steinbüchel, 2005). In several scl-PHA producing bacteria, the major phasin, designated PhaP, is negatively regulated by PhaR (also designated PhbF, PhaF or AniA), a DNA-binding regulator. The regulatory circuit in which PhaR participates has been well studied in *R*. *eutropha* and *P*. *denitrificans*. In these bacterial species, PhaR is able to bind to three different targets: the promoter region of *phaP*, the promoter region of its own gene, and the surface of the granules, so this regulator is a granule associated protein. It is proposed that when bacteria are not accumulating PHAs, the expression of *phaP* and *phaR* remains at basal level. In this scenario, the presence of PhaP is not needed and its expression is repressed by direct binding of PhaR to the *phaP* promoter region. The excessive production of PhaR is also repressed by autoregulation. Under favorable PHA-synthesis conditions, the cells start to accumulate PHAs and PhaR dissociates from DNA and binds to the new polymer chains, allowing the expression of *phaP*. As the PHAs synthesis continues, the granules enlarge in size, and their surfaces become covered with PhaP. If PHAs synthesis stops or degradation occurs predominantly, PhaR molecules are freed and bind to the upstream elements of both *phaP* and *phaR*, repressing their expression (Maehara et al., 1999, 2002; York et al., 2002; Pötter et al., 2002, 2005).

PhaR is also a repressor of *phaP* gene in the purple, non-sulfur bacterium *R*. *sphaeroides* FJ1, although it is not known if the regulatory mechanism is the same. It is expected to be similar, because *R*. *sphaeroides* PhaR binds to the promoter regions of *phaP* and *phaR*, repressing their expression (Chou et al., 2009; Chou and Yang, 2010).

Homologs to PhaR (also known as *aniA* for anaerobically-induced gene A) were identified in rhizobia species, such as *S*. *meliloti* (Povolo and Casella, 2000); *R*. *etli* (Encarnación et al., 2002) and *Bradyrhizobium diazoefficiens* (Quelas et al., 2016). In *R*. *etli*, PhaR (AniA) is necessary for PHB synthesis, because a *phaR*:Tn5 mutant produced only around 40% the PHB level of the wild type; moreover, *phaR* inactivation caused a profound modification of global protein expression, which includes the disappearance of PhaB in proteome maps (Encarnación et al., 2002), suggesting that PhaR plays a more global regulatory role.

Even when in all these bacteria PhaR is not a direct PHA-synthesis regulator, knowledge about the regulation of expression of phasins might be important for establishing optimal conditions for PHAs production, both in natural PHA-producers or in recombinant bacteria. However, in *H*. *seropedicae* SmR1 PhaR has a wider role in the control of PHAs metabolism, because it binds to the regulatory regions of eleven genes, which include the phasin gene *phaP*1, but also the *pha* biosynthetic genes, acting on them as a repressor (Kadowaki et al., 2011). Surprisingly, the inactivation of *phaR* reduced PHB production 3.2-fold instead of increasing it, suggesting a role in the regulation of other genes important for PHB accumulation (Batista et al., 2016).

##### PhaF from Pseudomonas spp.

Two major granule-associated proteins, PhaF and PhaI, are found in the mcl-PHA producer *P*. *aeruginosa* GPo1 (Prieto et al., 1999). PhaF is a histone-H1-like protein, with two different domains, a DNA-binding domain at the C-terminus, and a phasin domain for PHA-binding, at the N-terminus. Although PhaF shares these bifunctional characteristics with PhaR from the scl-PHA producers (also named PhbF), the amino acid sequence of PhaF or PhaI showed no similarity to PhaR (Maehara et al., 2002); therefore, they are two different proteins with different functions. Early characterization of PhaF suggested that it represses the expression of *phaC*1 synthase, *phaI* and its own expression, because *phaF* mutants showed increased transcription of the *pha* cluster. The model proposed that PhaF could bind DNA, repressing the expression of *phaC*1 and *phaIF* operon, similarly to PhaR (Prieto et al., 1999). A more recent study showed that PhaF binds DNA in a non-specific manner and is involved in the segregation of granules between daughter cells during cell division (Galán et al., 2011).

**FIGURE 1:**
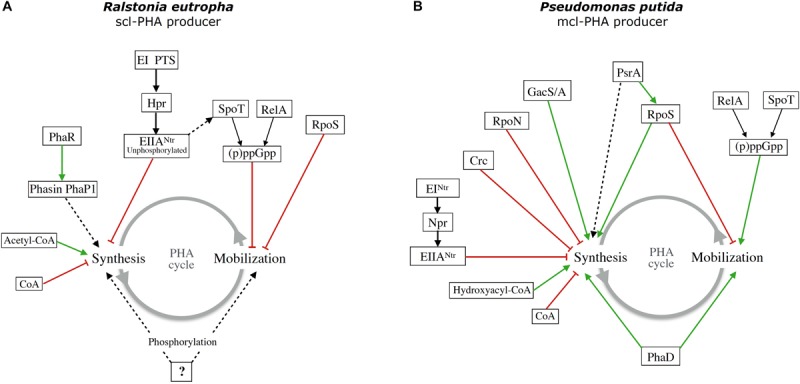
Schematic diagram of the regulation of PHAs metabolism by global and specific regulatory systems in two model organisms: **(A)** the scl-PHA producer *Ralstonia eutropha* and **(B)** the mcl-PHA producer *Pseudomonas putida* KT2440. Green arrows indicate positive regulation and red lines negative regulation or effect. Dashed lines indicate unknown intermediates and unknown mechanism of regulation.

As can be appreciated, the regulatory mechanisms implicated in the control of PHAs metabolism are diverse and can be different in different organisms. Most of them have not been fully characterized. In Figure 1, diagrams summarizing what is known in two different model organisms for PHAs production are shown.

## Regulation of Phb Synthesis in *Azotobacter*

*Azotobacter vinelandii* is an scl-PHAs producing bacterium that synthesizes mainly PHB, although it can synthesize P(3HB-co-3HV) when valerate, heptanoate or nonanoate are added to the growth medium (Page et al., 1992). *A*. *vinelandii* can accumulate PHB up to 80% of the cell dry weight (Millán et al., 2017), and the polymer produced has a high molecular weight (6.6 × 10^6^ Da) (Castillo et al., 2017). This bacterium can grow and produce PHB on several substrates, including organic acids, alcohols, sugars (Noar and Bruno-Bárcena, 2018) as well as low-cost substrates, like cane or beet molasses, corn syrup, malt extract, fish peptone and olive mill wastewater (Page, 1989, 1992a,b; Page et al., 1992; Page and Cornish, 1993; Chen and Page, 1994, 1997; Cho et al., 2001; Cerrone et al., 2010).

The allosteric regulation of the biosynthetic enzymes was reported in a species of this genus (*A*. *beijerinckii*) by Senior and Dawes (1973). This regulation was later confirmed to be similar in *A*. *vinelandii* UWD, where free CoA negatively regulated 3-ketothiolase, but this inhibition was overcome by acetyl-CoA (Manchak and Page, 1994).

**FIGURE 2:**
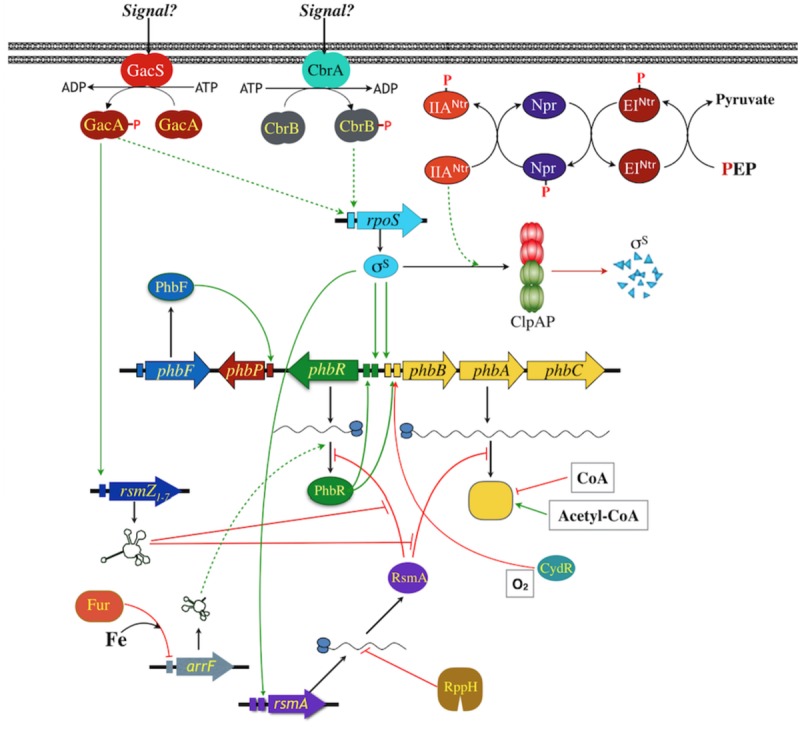
Model for the regulation of *phbBAC* gene expression in *A*. *vinelandii*. Promoters are indicated as colored rectangles, two of them indicate two promoters identified. Positive regulation is indicated with green lines; red lines indicate negative regulation; dashed lines indicate unknown intermediates or mechanism.

Many details are known about the molecular genetics of PHAs production in *A*. *vinelandii* (Figure 2). The biosynthetic operon *phbBAC*, encoding the enzymes for PHAs synthesis has been characterized (Segura et al., 2000, 2003; Peralta-Gil et al., 2002). As was described previously, the *phbR* gene, encoding a transcriptional activator of the AraC family named PhbR, was identified upstream and in the opposite direction to the *phbBAC* operon. Inactivation of *phbR* reduced PHB accumulation and transcription of *phbBAC*. This operon is transcribed from two overlapping promoters (PB1 and PB2), where the −35 region of PB1 overlaps the −10 of PB2. PhbR was shown to bind specifically to a couple of almost identical 18 bp sites present in the *phbBAC* promoter region, and transcription from pB1 was shown to be activated by PhbR, whereas transcription from PB2 was shown to depend on RpoS sigma factor (Peralta-Gil et al., 2002; Hernández-Eligio et al., 2011).

With respect to transcription of *phbR*, it is also initiated from two promoters, pR1 and pR2 (Peralta-Gil et al., 2002). Transcription from pR2 depends on RpoS (Hernández-Eligio et al., 2011). By using a *phbR*:*gusA* gene fusion it was shown that transcription of *phbR* is higher in the presence of a PhbR wild type copy, revealing a role for PhbR as auto-activator (Hernández-Eligio et al., 2011).

In addition to the regulation by PhbR and RpoS, the GacS-GacA two-component global regulatory system was shown to control PHB synthesis, as well as the production of other lipids and the polysaccharide alginate (Castañeda et al., 2000; Manzo et al., 2011; Romero et al., 2016; Trejo et al., 2017). The GacS-GacA system, formed by the GacS sensor kinase and the response regulator GacA, is conserved in gram-negative bacteria, and in the *Pseudomonadaceae* family, to which *Azotobacter* genus belongs, where it controls the expression of genes involved in secondary metabolism, phyto-pathogenesis and QS, among other processes. The regulation by GacS-GacA is mediated through a pathway known as Gac-Rsm (Lapouge et al., 2008), where GacA homologs activate the transcription of small regulatory RNAs named RsmZXY, that interact with a small protein named RsmA. The RsmA protein acts as a translational repressor through binding to its target mRNAs. Therefore, the interaction of RsmA with the RsmZXY RNAs counteracts its repressor activity.

Regarding the regulation of PHB synthesis in *A*. *vinelandii* by this system, it was first reported that mutations in either *gacS* or *gacA* genes impaired synthesis of this polymer (Castañeda et al., 2000, 2001), and later, that the Gac-Rsm pathway in *A*. *vinelandii* was composed by one RsmA protein and eight non-coding small RNAs, named RsmZ1–7 and RsmY (Manzo et al., 2011; Hernández-Eligio et al., 2012). Transcription of the *rsm* RNAs was shown to be dependent on GacA (Manzo et al., 2011; Hernández-Eligio et al., 2012) and at least two of these RNAs, RsmZ1, and RsmZ2, were shown to specifically bind RsmA (Manzo et al., 2011; Hernández-Eligio et al., 2012). Inactivation of *rsmA* increased PHB production and the RsmA protein was also demonstrated to bind to the predicted sites at the 5′ ends of the *phbR* and *phbBAC* mRNAs. Thus, RsmA binding to these transcripts represses their translation and also negatively affect their stability, and at least RsmZ1 and RsmZ2, counteract this repression (Hernández-Eligio et al., 2012).

Besides the control of RsmA activity through binding to the *rsm* RNAs, its expression is also regulated. RsmA expression in *A*. *vinelandii* is controlled both transcriptionally and post-transcriptionally. The CbrA/CbrB and Crc/Hfq regulatory systems participate in the phenomenon of carbon catabolite repression (CCR) in *Pseudomonas* spp. and *A*. *vinelandii* (Quiroz-Rocha et al., 2017b; Martínez-Valenzuela et al., 2018; for a recent review see Bharwad and Rajkumar, 2019). Inactivation of *cbrB* resulted in a reduction of the *rsmA* mRNA levels. The effect of CbrB on *rsmA* expression was proposed to be through the control of RpoS expression, because CbrA-CbrB is required for optimal levels of RpoS, and one of the promoters transcribing *rsmA* is RpoS dependent (Quiroz-Rocha et al., 2017a). However, the mechanism by which CbrB affects RpoS is still unknown.

With respect to the post-transcriptional control of RsmA expression, it was reported that its transcript is a substrate of an mRNA degradation pathway in which the pyrophosphohydrolase enzyme RppH participates (Bedoya-Pérez et al., 2018). Inactivation of *rppH* resulted in significantly lower levels of PHB, through a reduction in the expression of PhbR (at the translational level). This effect is through RsmA, because inactivation of its gene in the *rppH* mutant restored *phbR* expression and PHB synthesis, due to an increase in the level and stability of the *rsmA* transcript (Bedoya-Pérez et al., 2018). Thus, in summary, and as shown in Figure 2, GacA activates transcription of RsmZ RNAs, these RNAs bind to RsmA to prevent its binding to its target mRNAs that include the *phbR* and the *phbBAC* transcripts. The expression of RsmA, on the other hand, is controlled by an RNA degradation pathway with the participation of RppH.

As mentioned above, another regulatory mechanism controlling the synthesis of PHB in *A*. *vinelandii* is the nitrogen-related phosphotransferase system (PTS^Ntr^; Figure 2). This is homologous to the carbohydrate PTS which in many bacteria transports and phosphorylates sugars and, in addition, participates in regulatory processes, such as carbon catabolite repression, chemotaxis, biofilm formation and virulence (Deutscher et al., 2014). PTS^Ntr^ is present in most gram-negative bacteria and is composed by EI^Ntr^, Npr and EIIA^Ntr^ proteins, encoded, respectively, by the *ptsP, ptsO* and *ptsN* genes. These components participate in a phosphorylation cascade starting from phosphoenolpyruvate, and ending with the final phospho-acceptor EIIA^Ntr^. The PTS^Ntr^ seems to be exclusively involved in regulatory functions (Pflüger-Grau and Görke, 2010). Another difference between carbohydrate PTS and PTS^Ntr^ is the existence of a GAF domain in the N-terminal of EI^Ntr^ that can bind diverse ligands. In *E*. *coli*, binding of glutamine or α-ketoglutarate to this domain allows the sense the cellular nitrogen status to modulate the phosphorylation of PTS^Ntr^ (Lee et al., 2013). In *A*. *vinelandii*, strains carrying a non-phosphorylated form EIIA^Ntr^, due to mutations in *ptsP*, *ptsO* or *ptsN*H56A (in which the phosphorylatable histidine residue was changed by an alanine), are unable to produce PHB and showed a reduction in transcription of *phbR* and *phbBAC* (Segura and Espín, 1998; Noguez et al., 2008).

When EIIA^Ntr^ is unphosphorylated, the stability of the RpoS, necessary for transcription of both *phbR* and *phbBAC*, is reduced. It was found that the chaperone-protease ClpAP complex is involved, because inactivation of their genes in strains carrying unphosphorylated EIIA^Ntr^, restored the levels and stability of RpoS, as well as the synthesis of PHB. Thus, in *A*. *vinelandii* the mechanism by which the unphosphorylated EIIA^Ntr^ controls gene expression includes the induction of RpoS degradation by the proteolytic complex ClpAP (Muriel-Millán et al., 2017).

A relationship between GacA and the PTS^Ntr^ system was recently revealed. In the wild-type strain of *A*. *vinelandii*, both phosphorylated and unphosphorylated forms of EIIA^Ntr^ are present. In contrast, in a *gacA* mutant only the unphosphorylated EIIA^Ntr^ was detected (Trejo et al., 2017). Thus, GacA somehow regulates the PTS^Ntr^ phosphorylation cascade, which in turn controls PHB synthesis at the transcriptional level. The mechanism for this regulation is unknown; however, the hypothesis is that the expression of genes under *gacA* control could affect the nitrogen status, modulating phosphorylation of PTS^Ntr^ through binding of glutamine and α-ketoglutarate to the EI^Ntr^ GAF domain.

PHB production in *A*. *vinelandii* is higher under oxygen limiting conditions, and the oxygen-responsive transcription factor CydR (Fnr-like), has been implicated in this control (Wu et al., 2001). Inactivation of the *cydR* gene, allowed a considerably higher accumulation of PHB than the wild type throughout the exponential growth phase. Proteomic analysis showed that the *cydR* mutant overexpressed the PHB biosynthetic enzymes β-ketothiolase and acetoacetyl-CoA reductase, and decreased expression of succinyl-CoA, an enzyme participating in PHB mobilization. Thus, CydR controls PHB metabolism, although the authors did not determine whether this control is direct or not. The behavior of this mutant strain under different oxygen conditions has not been tested, but it could be interesting to study for PHB production improvement.

Iron-limiting conditions also increase PHB accumulation in *A*. *vinelandii* (Page, 1982), and a regulatory small RNA ArrF has been found to participate (Pyla et al., 2009; Muriel-Millán et al., 2014), although the mechanism seems to differ depending on the strain. The Fur protein (ferric uptake repressor), a regulator of iron homeostasis, represses the expression of *arrF* in the presence of Fe(2+). In response to iron depletion, this repression is released and the expression of *arrF* is increased. ArrF, in turn, affects translation of its target mRNAs through the complementarity of its 5′-untranslated region, acting as antisense RNA (Jung and Kwon, 2008). In the *A*. *vinelandii* KCTC 23243, which produces small amounts of PHB, inactivation of *arrF* increased accumulation of the polymer 300-fold. Expression levels of the *phbBAC* biosynthetic genes and of their transcriptional activator *phbR* were also increased on this condition. ArrF was proposed to participate in the regulation by negatively controlling the expression of *phbR*, probably in an antisense manner (Pyla et al., 2009). In *A vinelandii* UW136, iron limitation also increases PHB accumulation and *phbBAC* transcription, through the post-transcriptional activation of *phbR* expression. However, in this strain, ArrF was shown to be required for full induction of the *phb* gene expression and PHB accumulation, both under iron-limited and non-lifted conditions. ArrF was concluded to act as a post-translational activator of *phbR*, thus activating transcription of *phbBAC* and production of PHB (Pyla et al., 2009; Muriel-Millán et al., 2014).

## Bioprocess in Bioreactors for High-Level Production of Phb and Modification of Molecular Mass, Using *A*. *Vinelandii* Regulatory Mutants

The main negative regulators identified in *A*. *vinelandii* so far are the small RsmA protein, that is a repressor of the translation of the *phbR* and *phbBAC* transcripts (Hernández-Eligio et al., 2012), and the Enzyme EIIA^Ntr^, a protein that in its unphosphorylated form promotes degradation of RpoS by the ClpAP chaperone protease complex (Muriel-Millán et al., 2017).

As a result of the above knowledge, *A*. *vinelandii* strains carrying mutations in *rsmA* and *ptsN*, the genes coding for two negative regulators were constructed. Strain OPN, which carries a mutation inactivating *ptsN*, showed a PHB overproducing phenotype when cultured on PY sucrose agar plates, reaching a 70% higher accumulation than the value reached using the OP strain under that condition. On the other hand, when grown in shake flasks with PY sucrose medium a slightly higher volumetric amount of PHB was obtained with strain OPN, reaching 4.1 g L^–1^ at 60 h of cultivation; whereas, in the case of the wild-type OP strain, the PHB content was of 3.5 g L^–1^. However, the specific production was considerably higher in the OPN mutant that reached 2.69 g PHB g protein^–1^, which was almost 80% higher than that observed for the OP wild-type strain (1.52 g PHB g protein^–1^) (Peña et al., 2013). This work also demonstrated that the molecular mass (MM) of the PHB was significantly influenced by *ptsN* mutation in addition to the aeration conditions. A higher MM was obtained under low-aeration conditions in both strains; however, a maximal molecular mass of 2,026 kDa was obtained with strain OPN, a value 2-fold higher than that obtained from the parental strain OP (MM = 1,013 kDa) under the same condition (Peña et al., 2013). From a technological point of view, the manipulation of the molecular weight of PHB by means of changes in the aeration conditions and the use of regulatory mutants is a convenient method that could considerably improve the properties of PHB.

Later, García et al. (2014) reported the use of *A*. *vinelandii* OPNA, a double mutant that carries inactivations on both PTS^Ntr^ and RsmA-RsmZ/Y regulatory systems. By using a strategy of exponential feeding coupled with nutrient pulses the production of PHB increased 7-fold (with respect to the batch culture) to reach a maximal PHB concentration of 27.3 ± 3.2 g L^–1^ at 60 h of growth.

With the use of strain OPNA, both in fed-batch and batch cultures, in addition to over-producing PHB, the synthesis of the polymer is growth associated and therefore it is not necessary to limit the culture (nutrients or oxygen) to promote the accumulation of PHB. This mutant is able to accumulate PHB up to 83% of its dry weight, even when grown under non-oxygen-limited conditions (4% of DOT). This behavior was clearly different from those reported for other *A*. *vinelandii* strains. For example, Flores et al. (2013) reported that in batch cultures of *A*. *vinelandii* ATCC 9046 at 5% of DOT, the cells accumulated only 20% of PHB (based on dry weight). In addition, Senior et al. (1972) reported PHB accumulation in *Azotobacter* under conditions of oxygen limitation, due to the increase of the ratios of NADPH + /NADP and acetyl-CoA/CoA, which promote PHB biosynthesis. Finally, an additional point to highlight is that the rates of oxygen consumption are 20% lower in the mutant than those observed with the parental strain. This offers advantages, from the operational point of view, because the commercial use of these strains would significantly reduce the aeration needs in large fermenters and therefore would decrease the production costs of the polymer, improving the balance of the economy of the process.

More recently, Castillo et al. (2017) and García et al. (2019) evaluated the use of OPNA mutant strain for PHB production at different scales. In both reports, the authors showed that by using this strain in batch and fed-batch cultures it is possible to obtain a polymer of ultra-high molecular weight. It should be highlighted that a polymer having a very high molecular mass considerably improves its mechanical properties, expanding its potential applications. Castillo et al. (2017) found that in 3.0 L batch and fed-batch cultures in a fermenter, using different carbon/nitrogen molar ratios (10, 14, and 18), the OPNA strain produced PHB of high and ultra-high weight average molecular weight, with values between 2.3 and 6.6 MDa. This was highly dependent on the initial carbon/nitrogen ratio, reaching the highest value (6.6 MDa) in cultures conducted with a ratio of 18 and the lowest (2.3) with a ratio of 10. In fed-batch cultures, using a two-pulse feeding strategy, it was possible to obtain a global PHB volumetric productivity of 0.56 g L^–1^ h^–1^ and polymer concentration of 27.6 g L^–1^.

García et al. (2019) scaled the process to fed-batch systems in 30 L fermenter using low-cost raw materials and found that the OPNA strain was able to reach good growth and PHB production, decreasing significantly the production cost of the polymer. When using the power input (P/V) as a criterion, it was possible to scale-up the process from 3 to 30 L bioreactors, confirming that the use of power input is an appropriate engineering parameter to scale-up the production process. In the 30 L bioreactor, both the polymer concentration and productivity were similar or even better than the values obtained in the fermenter of 3.0 L.

## Concluding Remarks and Future Prospects

In this article, the regulatory mechanisms that control PHAs metabolism are reviewed. PHAs accumulate in response to nutritional or environmental conditions. This is mediated by diverse regulatory mechanisms that may vary between different PHAs producing species.

The regulators of PHAs metabolism operate at different levels. The first one described was the control of the activity of the enzymes involved. This can be accomplished by allosterism, interaction with some metabolites, or through covalent modification of the biosynthetic or mobilizing enzymes. Also, many examples of transcriptional regulation by proteins like alternative sigma factors and transcription factors have been reported. Likewise, the control of transcription or translation of *pha* genes by small regulatory RNAs has been reported.

The phenotypes of mutants affected in these regulatory mechanisms have also been revised. Some of these regulatory mutants show PHAs overproducing phenotypes and even show modifications in the composition or molecular weight of the PHAs produced. Thus, knowledge of the regulatory mechanisms controlling PHAs metabolism can be used to design strains with better capabilities for the production of bioplastics. However, in many cases, the details of the mechanisms controlling *pha* gene expression are missing. In several examples, not even the targets of the regulation have been identified and only the final effect on PHAs accumulation is reported. Therefore, a deeper characterization of the regulatory systems reported would be useful.

Among the regulators participating in the control of PHAs metabolism, there are global regulators and also PHAs specific regulators. In many of the examples here reviewed it is shown that the need for nutrient deprivation to accumulate PHAs is mediated by some of these global regulators because their inactivation eliminates this limitation and the polymers can be accumulated during exponential growth under nutrient replete conditions. Some of these mutants even show other metabolic changes favorable for PHA production, contributing to the improved production phenotypes observed.

A better understanding of the fine details of the specific regulatory mechanisms that control PHAs accumulation could help identify targets for modification to improve polymer productivity, but a wider comprehension of the global regulatory networks connecting PHAs metabolism with the rest of the metabolism would also be important for this purpose. This can be achieved through whole-transcriptome or proteome studies of regulatory mutants because these are powerful tools for the study of wide responses. For example, deep RNA-sequencing analysis of these mutants would reveal not only the *pha* genes regulated, but other metabolic pathways which are co-regulated and probably related with PHAs metabolism, helping in the identification of genetic modifications for strain improvement.

The case of *A*. *vinelandii*, an organism whose PHB regulatory network has been extensively studied, illustrates how the modification of some regulatory elements (inactivation of two negative regulators), can be useful for the improvement of polymer production. Similar strategies could be used to explore the production capacities of other PHAs producing bacteria.

With respect to the control of the balance between PHAs synthesis and degradation, more research is needed to understand what conditions or effectors determine if the polymer is accumulated or mobilized, especially considering that the simultaneous expression of PHA synthase and depolymerases has been reported in some organisms (Doi et al., 1990; Ren et al., 2009; Brigham et al., 2012). From a practical point of view, this balance between PHA synthesis and depolymerization is relevant because it affects the amount and characteristics of the polymers produced. Low depolymerizing activities allow to increase PHA production and also favor the synthesis of ultra-high molecular weight PHAs, which in turn affects the properties of the polymer (Arikawa et al., 2016; Adaya et al., 2018).

## Author Contributions

DS contributed conception of the study. All authors wrote the sections of the manuscript, contributed to manuscript revision, read and approved the submitted version.

## Conflict of Interest

The authors declare that the research was conducted in the absence of any commercial or financial relationships that could be construed as a potential conflict of interest.
